# Efficacy of different storage methods and duration of blood samples on biochemical indexes in healthy blood donors

**DOI:** 10.12669/pjms.41.7.10873

**Published:** 2025-07

**Authors:** Yu Wang, Aijun Liu, Chao Li, Junpeng Zhao, Maqian Su, Zeyu Wang

**Affiliations:** 1 Yu Wang Department of Quality Control, Tangshan Blood Stations, Tangshan 063000, Hebei, China; 2 Aijun Liu Department of Quality Control, Tangshan Blood Stations, Tangshan 063000, Hebei, China; 3 Chao Li Department of Hematology and Oncology, Beijing Aerospace General Hospital, Beijing 100000, Beijing, China; 4 Junpeng Zhao Tangshan Food and Drug Comprehensive Inspection, Testing Center, Tangshan 063000, Hebei, China; 5 Maqian Su Blood Group Research Laboratory, Tangshan Blood Stations, Tangshan 063000, Hebei, China; 6 Zeyu Wang Department of 1^st^ Blood Collection, Tangshan Blood Stations, Tangshan 063000, Hebei, China

**Keywords:** Blood Samples, Biochemical Indexes, Storage Duration, Storage Method

## Abstract

**Objective:**

To explore the efficacy of different blood specimen preservation methods and time on biochemical indicators of healthy blood donors.

**Method:**

This was a retrospective observational study. Two hundred healthy blood donors were selected as the study participants at Beijing Aerospace General Hospital from April 2023 to September 2023. According to the preservation method of blood samples, they were divided into three groups: ABC, Group-A was stored at room temperature (20°C), Group-B was stored at -20°C, and Group-C was stored at 4°C. Compared and analyzed the differences in biochemical indicators between different time periods and levels within one hour.

**Result:**

The results of various test indexes were measured within one hour, The levels of ALT, CK, LDH, GLU, Cr, and UA in Group-A changed after 12 h, AST and BUN changed after 24 hour, and ALB values changed significantly after one week (p<0.05); The GLU of Group-B changed after 12 hours, and the Cr changed after 24 hours, ALT, AST, CK, LDH, BUN, and UA levels changed after one week (p<0.05), while ALB levels remained unchanged (p=0.23); The video of ALT and LDH in Group-C showed changes after 24 hours, while the values of other indicators changed after one week (p<0.05).

**Conclusion:**

Under normal temperature storage, most indicators change within 12-24 hours. Storage at 4°C can delay the changes of some indicators, while -20°C is more suitable for short-term stable storage.

## INTRODUCTION

Blood is the essential component of the human body, the composition of which may be changed to varying degrees during the onset of various diseases, and testing blood biochemical indexes facilitates a better understanding of the physical condition of patients.^1^ Blood tests are commonly used in clinical diagnostics, and the mechanisms, conditions, and severity of diseases can be investigated through the analysis of blood samples.^2^ This is especially true for many latent diseases, where treatment and diagnosis often depend on the results of blood biochemical indexes, thereby providing essential references for the treatment of patients.^3^

In terms of quality control for clinical laboratories, pre-analytical quality control is the primary and crucial step, while proper handling of samples is vital for ensuring the reliability of test results, in which the storage of blood samples has always been a key focus in clinical blood testing, as the time and method of storage directly affect the accuracy of test results.^4^ Sousa et al.^5^ suggest that the storage of blood samples is an integral part of pre-analytical quality control in medical testing, and the storage time of blood samples exhibits varying degrees of impact on certain biochemical indexes. In this regard, errors due to the handling of samples should be minimized as much as possible to obtain accurate results for various tests with the widespread application of fully automated biochemical analyzers, traditional laboratory workflows have changed from testing different items with multiple tubes to multi-item testing with a single tube, gradually using the original tubes for testing with instruments.

This means that in some laboratories, especially those with a large number of samples, blood samples may not be analyzed promptly after collection, and in special cases, some samples may be stored for extended periods of time, even overnight. Therefore, it remains to be investigated whether biochemical indexes change in blood samples stored for extended periods of time using different storage methods. Given this, healthy blood donors were selected as the study participants to investigate the effects of different storage methods and duration of blood samples on their biochemical indexes.

## METHODS

This was a retrospective observational study. Two hundred healthy blood donors were selected as the study participants at Beijing Aerospace General Hospital from April 2023 to September 2023. This study used G-power 3.1.9.2 software to calculate the required sample size,α=0.05, under the condition of a statistical test power of 1-α=0.95, a sample size of 180 cases was obtained to prevent an increase of 10% in dropout among the research subjects. Ultimately, the total number of cases in this study was determined to be 200. The participants in this Group-Consisted of 106 males and 94 females, and the youngest and oldest were aged 22 and 43 years, respectively, with a mean age of (32.57±3.68) years.

### Ethical approval:

The study was approved by the Institutional Ethics Committee of Tangshan Blood Stations (No.: 2023062001; Date: June 20, 2023), and written informed consent was obtained from all participants.

### Inclusion criteria:

- Healthy individuals undergoing physical examinations;
- Aged 18~60 years old;
- Liquid samples are free from phenomena such as lipid turbidity and jaundice;
- Blood sample collection process does not involve interference factors such as poor blood flow or oscillation.

### Exclusion criteria:

- Participants with severe organic diseases and mental diseases during physical examinations were excluded;
- Pregnant or lactating women;
- whose blood samples do not meet the study criteria.

### Test Methods:

Venous blood samples were collected from all participants under a fasting state, with various test indexes within one hour after blood collection used as controls. According to the storage method, blood samples were divided into the A, B, and C groups, in which Group-A was stored at room temperature (20°C); Group-B was stored and frozen at -20°C; Group-C was stored at 4°C in a refrigerator. Afterward, biochemical indexes, such as alanine aminotransferase (ALT), aspartate aminotransferase (AST), creatine kinase (CK), lactate dehydrogenase (LDH), glucose (GLU), serum albumin (ALB), blood urea nitrogen (BUN), creatinine (Cr), and uric acid (UA) were measured in the three groups using an automatic biochemical analyzer after 12 hours, 24 hours and one week.

### Statistical Analysis:

SPSS 20.0 software was used for the statistical analysis of all data, and measurement data were expressed as (*χ̅*±*S*). Repeated measures analysis of variance was utilized for comparing the test data, and pairwise comparisons were conducted using the LSD-t test. P < 0.05 was considered statistically significant.

## RESULTS

The analysis of biochemical indexes at different time points for the blood samples stored at room temperature (Table-I) suggested that as the storage duration extended, biochemical indexes such as ALT, AST, CK, LDH, GLU, ALB, BUN, Cr, and UA significantly changed compared with the values measured within one hour, and the differences were statistically significant (p<0.05). Among them, ALT, CK, LDH, GLU, Cr, and UA values changed significantly at 12 hours, 24 hours, and one week compared with those measured at one hour, AST and BUN values changed significantly after 24 hours, while ALB values changed significantly after one week.

**Table-I:** Analysis of Differences in Biochemical Indexes at Different Time Points in Group-A(*χ̅*±*S*), n=200.

Index	1h	12h	24h	After 1W	F	p
ALT(U/L)	20.43±5.08	17.31±5.10*	14.08±4.33*	11.32±3.17*	8.49	0.00
AST(U/L)	42.35±11.87	43.79±11.46	45.82±11.32*	47.11±12.04*	3.27	0.00
CK(U/L)	72.26±18.38	68.26±17.35*	66.73±17.92*	65.17±15.80*	2.28	0.02
LDH(U/L)	175.04±30.71	182.57±31.47*	185.09±30.83*	194.26±28.06*	2.33	0.02
GLU(mmol/L)	5.28±2.16	4.84±1.78*	4.10±2.03*	3.36±1.68*	2.47	0.01
ALB(U/L)	42.91±2.85	42.27±2.78	42.18±2.64	38.76±3.07*	2.27	0.02
BUN(mmol/L)	5.72±1.23	5.98±2.00	6.76±1.68*	7.45±2.14*	6.24	0.00
Cr(μmol/L)	74.52±7.89	76.09±8.02*	82.18±7.94*	85.23±8.84*	2.67	0.01
UA(μmol/L)	332.63±28.35	343.54±26.57*	351.82±25.47*	368.04±23.70*	4.07	0.00

* P<0.05

With the prolonged storage duration for blood samples stored and frozen at -20°C, biochemical indexes, including ALT, AST, CK, LDH, GLU, BUN, Cr, and UA, significantly changed compared with those measured within one hour, and the differences were statistically significant (p<0.05). Among them, ALT, AST, CK, LDH, BUN, and UA values changed after one week, GLU values changed after 12 hours, and Cr values changed after 24 hours, with no significant changes in ALB values observed at any time point (p=0.23) (Table-II).

**Table-II:** Analysis of Differences in Biochemical Indexes at Different Time Points in Group-B (*χ̅*±*S*) n=200.

Index	1h	12h	24h	After 1W	F	p
ALT(U/L)	20.43±5.08	20.32±5.06	20.40±5.63	17.08±4.72*	5.43	0.00
AST(U/L)	42.35±11.87	42.60±12.13	42.68±11.82	45.30±11.42*	2.73	0.00
CK(U/L)	72.26±18.38	71.89±17.53	71.59±17.39	66.73±17.36*	2.94	0.00
LDH(U/L)	175.04±30.71	176.38±31.07	175.32±31.34	185.46±30.28*	3.28	0.00
GLU(mmol/L)	5.28±2.16	4.71±2.03*	4.26±1.74*	3.36±1.77*	5.12	0.00
ALB(U/L)	42.91±2.85	42.63±2.21	42.86±2.46	42.57±2.08	1.27	0.23
BUN(mmol/L)	5.72±1.23	5.73±1.68	5.72±1.25	6.48±2.21*	4.43	0.00
Cr(μmol/L)	74.52±7.89	75.12±7.95	79.83±7.56*	83.63±7.92*	5.32	0.00
UA(μmol/L)	332.63±28.35	335.32±27.62	336.80±26.16	345.56±25.44*	3.60	0.00

* P<0.05

As the storage duration extended for blood samples stored at 4°C in a refrigerator, biochemical indexes such as ALT, AST, CK, ALB, LDH, GLU, BUN, Cr, and UA changed significantly compared with those measured within one hour, and the differences were statistically significant (p<0.05). Among them, ALT and LDH values changed after 24 hours, and the values of other indexes changed after one week (Table-III).

**Table-III:** Analysis of Differences in Biochemical Indexes at Different Time Points in Group-C (*χ̅*±*S*) n=200.

Index	1h	12h	24h	After 1W	F	p
ALT(U/L)	20.43±5.08	20.75±5.47	16.68±5.37*	16.27±5.35*	4.31	0.00
AST(U/L)	42.35±11.87	42.46±11.73	42.54±12.60	46.47±11.18*	3.65	0.00
CK(U/L)	72.26±18.38	72.06±18.48	72.63±18.83	68.40±18.61*	5.02	0.00
LDH(U/L)	175.04±30.71	175.42±26.54	178.68±28.57*	184.36±31.55*	2.16	0.02
GLU(mmol/L)	5.28±2.16	5.35±2.42	4.31±2.04	3.26±1.53*	5.53	0.00
ALB(U/L)	42.91±2.85	42.60±2.24	42.53±2.18	46.61±3.21*	3.75	0.00
BUN(mmol/L)	5.72±1.23	5.48±1.25	5.50±1.64	6.83±2.04*	3.14	0.00
Cr(μmol/L)	74.52±7.89	74.63±7.39	74.92±7.25	82.57±7.31*	5.87	0.00
UA(μmol/L)	332.63±28.35	334.56±26.74	332.56±26.80	343.68±27.64*	6.31	0.00

p<0.05

## DISCUSSION

This study investigated the effects of different storage conditions (room temperature, 4°C refrigeration, and -20°C freezing) and storage times on multiple biochemical indicators of blood samples. The results showed that with the extension of storage time, ALT, AST, CK, LDH, GLU, ALB, BUN, Cr, UA and other indicators all underwent varying degrees of changes, and the impact of different storage methods on the stability of biochemical indicators varied. These findings provide important references for clinical laboratory specimen processing and quality control before analysis. This study observed that under normal temperature (20°C) storage conditions, ALT, CK, LDH, GLU, Cr, and UA showed significant changes after 12 hours, while AST and BUN showed more significant changes after 24 hours, and ALB showed significant changes after one week. The preservation time and temperature of blood samples have a significant impact on the stability of biochemical indicators, which is consistent with multiple research findings.^6^-^10^ In specimens stored at 4°C, ALT and LDH showed significant changes after 24 hours, while the changes in other indicators (AST, CK, GLU, ALB, BUN, Cr, UA) mainly occurred after one week. This result is partially consistent with the recommendations of the Clinical Laboratory Standards Institute (CLSI) guidelines,^11^ which suggest that short-term storage at 4°C (<24 hours) is suitable for most biochemical tests, but long-term refrigeration may still lead to instability of some indicators. However, compared to some studies,^12^,^13^ the reduction rate of GLU in this study was slower, possibly due to the use of anticoagulants (such as sodium fluoride) or differences in specimen centrifugation time. Under the freezing storage condition of -20°C, GLU significantly decreased after 12 hours, Cr changed after 24 hours, while changes in ALT, AST, CK, LDH, BUN, and UA mainly occurred after one week, while ALB remained relatively stable. This is similar to multiple research findings,^14^,^15^ which suggest that short-term cryopreservation (<24 hours) has little effect on most biochemical indicators, but long-term freezing may still lead to a decrease in enzyme activity or metabolite degradation. However, unlike this study, some studies reported that cryopreservation may affect indicators such as LDH and K+.^16^ However, in this study, LDH only showed a significant increase after one week, which may suggest the influence of different laboratory treatment methods (such as freeze-thaw rate) on the results.

This study systematically compared the effects of different storage conditions and times on multiple biochemical indicators, further refining the stability changes of each indicator under different conditions. The main findings include significant changes in ALT, GLU, Cr and other indicators within 12 hours, suggesting that clinical laboratories should try to avoid storing specimens at room temperature for extended periods of time, especially for emergency or rapid testing projects.^17^ Storing at 4°C can delay changes in most indicators and is suitable for short-term (<24 hours) storage. However, ALT and LDH may still undergo significant changes after 24 hours, indicating that laboratories need to optimize the detection priority of refrigerated specimens based on testing items. Although freezing at -20°C can delay changes in some indicators (such as ALB), long-term freezing may still lead to significant changes in enzyme activity (ALT, AST) or metabolites (UA, Cr).^18^,^19^ Therefore, caution should be exercised when interpreting frozen specimens for retrospective studies.^20^ Based on the results of this study, we believe that the laboratory should arrange the testing sequence reasonably according to the stability of the testing items, such as prioritizing the detection of indicators such as GLU and ALT that are easily affected by time. For specimens that cannot be immediately detected, it is recommended to use short-term refrigeration at 4°C or packaged freezing storage, and clarify the maximum allowable storage time for different items.

### Limitations:

However, this study comes with the limitation of a small sample size. Despite various methods being available for blood sample storage in clinical practice due to advancements in techniques, this study only selected a few of the most commonly used methods for comparison without investigating and testing some advanced storage methods. As our in-depth research continues in the future, the sample size shall be increased while including new storage methods, so as to research in a more objective and comprehensive way.

## CONCLUSIONS

The biochemical test results of blood samples are closely related to storage conditions and time. Under normal temperature storage, most indicators change within 12-24 hours. Storage at 4°C can delay the changes of some indicators, while -20°C is more suitable for short-term stable storage. In clinical practice, personalized specimen preservation should be developed based on the characteristics of the testing items to ensure the accuracy of the test results.

### Authors’ Contributions:

**YW** and **AL:** Design and carried out the studies, analyzed of result. Responsible and accountable for accuracy and integrity of the work.

**CL and JZ:** Carried out the studies, literature search. Helped in the interpretation and analysis of results.

**MS** and **ZW:** Suggested the research project, prepared the original manuscript.

All authors discussed the results and revised the manuscript critically for important intellectual content.
